# Simulative Analysis of Stimulated Raman Scattering Effects on WDM-PON Based 5G Fronthaul Networks

**DOI:** 10.3390/s25103237

**Published:** 2025-05-21

**Authors:** Yan Xu, Shuai Wang, Asad Saleem

**Affiliations:** 1 School of Opto-Electronic Engineering, Zaozhuang University, Zaozhuang 277160, China; zzuxuy@163.com; 2 Collaborative Research Center, Shanghai University of Medicine & Health Science, Shanghai 201318, China

**Keywords:** 5G fronthaul networks, WDM-PON, stimulated Raman scattering (SRS), power changes, modulation formats

## Abstract

In future hybrid fiber and radio access networks, wavelength division multiplexing passive optical networks (WDM-PON) based fifth-generation (5G) fronthaul systems are anticipated to coexist with current protocols, potentially leading to non-linearity impairment due to stimulated Raman scattering (SRS). To meet the loss budget requirements of 5G fronthaul networks, this paper investigates the power changes induced by SRS in WDM-PON based 5G fronthaul systems. The study examines wavelength allocation schemes utilizing both the C-band and O-band, with modulation formats including non-return-to-zero (NRZ), optical double-binary (ODB), and four-level pulse amplitude modulation (PAM4). Simulation results indicate that SRS non-linearity impairment causes a power depletion of 1.3 dB in the 20 km C-band link scenario, regardless of whether the modulation formats are 25 Gb/s or 50 Gb/s NRZ, ODB, and PAM4, indicating that the SRS-induced power changes are largely independent of both modulation formats and modulation rates. This effect occurs when only the upstream and downstream wavelengths of the 5G fronthaul are broadcast. However, when the 5G fronthaul wavelengths coexist with previous protocols, the maximum power depletion increases significantly to 10.1 dB. In the O-band scenario, the SRS-induced maximum power depletion reaches 1.5 dB with NRZ, ODB, and PAM4 modulation formats at both 25 Gb/s and 50 Gb/s. Based on these analyses, the SRS non-linearity impairment shall be fully considered when planning the wavelengths for 5G fronthaul transmission.

## 1. Introduction

Nowadays, fifth-generation (5G) mobile communication technology (5G) is commercially available and provides notable enhancements in capacity, flexibility, energy efficiency, and end-to-end latency [1]. In comparison to the first generation (1G) to fourth generation (4G), 5G enables 1000 times more data and connected devices, 100 times quicker user data transfer rates, 10 times longer battery life, and five times lower end-to-end latency [2]. In recent years, there has been a significant rise in both the number of mobile subscribers and the pace of technological advancements. By 2025, mobile network services are projected to reach 6.5 billion people globally, with 40 billion personal smart devices and 100 billion worldwide connections [3]. Therefore, in response to the growing demands for high capacity and speed, cloud/centralized radio access network (C-RAN) has recently been proposed for boosting the transmission capacity and lowering the expenses of network operation. C-RAN is composed of centralized unit (CU), distribution unit (DU) and active antenna unit (AAU) [4]. The fronthaul network is defined as the link between DU and AAU. For the 5G fronthaul network bearer schemes, currently, optical fiber direct connection [5], passive wavelength division multiplexing (WDM) [6], active WDM/optical transport network (OTN) [6], and wavelength division multiplexing passive optical network (WDM-PON) are taken into account [7]. WDM-PON uses WDM technology to transmit multiple wavelengths through a single optical fiber, providing users with highly reliable and transparent transmission. In addition, WDM-PON can also save fiber resources, reduce costs, and facilitate the integration of mobile and fixed networks [8]. Therefore, the WDM-PON based 5G fronthaul networks have been intensively studied in the literature. For the wavelength allocation schemes, C-band has the strengths of low fiber attenuation and high commercialization [9], and O-band has the advantages of low dispersion coefficient. Hence, both C-band and O-band are potential solutions. For the modulation formats, non-return-to-zero (NRZ), optical double-binary (ODB), and four-level pulse amplitude modulation (PAM4) are three main advanced modulation techniques for achieving the speed of 25 Gb/s or even 50 Gb/s [10]. As a result, NRZ, ODB, and PAM4 modulation formats are all taken into consideration.

Contrary to expectations, stimulated Raman scattering (SRS) would occur when the upstream and downstream wavelengths of 5G fronthaul networks simultaneously pass through the optical fiber. Additionally, in future hybrid fiber and radio access networks, the 5G fronthaul wavelengths will share the same fiber with earlier protocols, making the impact of SRS even more pronounced. SRS refers to the strong interaction between laser and material molecules, giving the scattering process characteristics similar to stimulated emission. In WDM systems, the longer wavelengths act as Stokes wavelengths, while shorter wavelengths serve as pump wavelengths. Thus, the performance of the WDM systems is then significantly impacted by the power changes that result from the energy of the pump wavelengths transferring in part to the Stokes wavelengths. For the investigation of SRS non-linearity impairment in PON networks, some work has been carried out in literature. In [11,12], due to the effect of SRS, a power depletion of 4 dB was generated on GPON signal. J. Li et al. studied the SRS power depletion between the NG-PON2 and fronthaul signals [13]. Moreover, J. Li et al. studied the SRS-induced BER penalty when multiple TWDM downstream wavelengths in L-band and multiple fronthaul wavelength channels in C-band are simultaneously transmitted in optical fiber [14]. Y. Xu et al. investigated the SRS effect on upgraded Super-PON and NG-EPON systems [15,16]. As future hybrid fiber and radio access networks integrate 5G fronthaul with additional TWDM-PON technologies (such as Super-PON and NG-EPON) [17,18,19], the impact of SRS becomes increasingly severe, potentially compromising the loss budget of both 5G fronthaul and PON networks. This growing issue calls for detailed study.

In this paper, the power changes caused by SRS non-linearity impairment in 5G fronthaul networks based on WDM-PON are studied. When only the C-band upstream and downstream wavelengths of the 5G fronthaul are transmitted and 25 Gb/s or 50 Gb/s NRZ, ODB and PAM4 modulation formats are applied, the SRS-induced power depletion is 1.3 dB. As the number of simultaneously transmitted wavelengths increases, the SRS-induced power depletion rises to 10.1 dB, significantly impacting the loss budget of 5G fronthaul networks. In contrast, when the 5G fronthaul networks utilize the O-band for transmission, the power depletion is measured as 1.5 dB with 25 Gb/s or 50 Gb/s NRZ, ODB and PAM4 modulation formats.

## 2. Theoretical Analysis of SRS Non-Linearity Impairment

SRS is a third-order nonlinear optical process where a pump wave (*ω_p_*) interacts with a Stokes wave (*ω_s_*) through molecular vibrations (*Ω_v_*). Firstly, the SRS gain spectrum is given here. The energy conservation requires [20,21]:

(1) $$\omega_{p}=\omega_{s}+\Omega_{v}$$

This paper derives the SRS gain spectrum *g_s_*(*ω*) from first principles, combining quantum mechanical transitions and classical nonlinear optics.

The Raman effect arises from the modulation of the molecular polarizability *α* by vibrational modes *Q*(*t*):

(2) $$\alpha \left(t\right)=\alpha_{0}+{\left(\frac{\partial \alpha }{\partial Q}\right)}_{0}Q\left(t\right)\;$$

where ${\left(\frac{\partial \alpha }{\partial Q}\right)}_{0}$ is the Raman susceptibility tensor. The vibrational coordinate *Q*(*t*) is driven by the beat frequency of the pump and Stokes fields:

(3) $$Q\left(t\right)=\frac{1}{2}(Q_{0}e^{i\left(\omega_{p}-\omega_{s}\right)t}+c.c.)$$

where *Q*_0_ proportional to the nonlinear polarization.

The total electric field *E* = *Ep* + *Es* induces a nonlinear polarization *P_NL_*:

(4) $$P_{NL}\approx N{\left(\frac{\partial \alpha }{\partial Q}\right)}_{0}Q\left(t\right)E$$

where *N* is the molecular number density, and *Ep* and *Es* denote the field amplitudes of the pump wave and Stokes wave, respectively. The Stokes component of *P_NL_* at the frequency *ω_s_* is:

(5) $${P_{NL}}^{\left(s\right)}\approx \frac{N}{2}{\left(\frac{\partial \alpha }{\partial Q}\right)}_{0}Q\left(0\right)E_{p}$$

Using the slowly varying envelope approximation, the Stokes field amplitude *Es* evolves as:

(6) $$\frac{dE_{s}}{dz}=\frac{i\omega_{s}}{2cn_{s}}{P_{NL}}^{\left(s\right)}$$

where *n_s_* is the refractive index at *ω_s_*. Substituting *Q*_0_ from the driven harmonic oscillator model:

(7) $$Q_{0}=\frac{{\left(\frac{\partial \alpha }{\partial Q}\right)}_{0}E_{p}E_{s}^{*}}{m_{e}({\Omega_{v}}^{2}-{\left(\omega_{p}-\omega_{s}\right)}^{2}-i\Gamma_{v}\left(\omega_{p}-\omega_{s}\right))}$$

where *m_e_* is the effective vibrational mass and *Γ_v_* is the damping rate.

The intensity *I_s_* ∝ ${\left|E_{s}\right|}^{2}$ satisfies:

(8) $$\frac{{dI}_{s}}{dz}=g_{s}I_{p}I_{s}$$

where the SRS gain coefficient *g*(*ω*) is derived as:

(9) $$g\left(\omega \right)=\frac{\omega_{s}N}{c^{2}n_{p}n_{s}\varepsilon_{0}m_{e}\Omega_{v}}{\left|{\left(\frac{\partial \alpha }{\partial Q}\right)}_{0}\right|}^{2}\frac{\Gamma_{v}}{{{(\Omega }_{v}-\left(\omega_{p}-\omega_{s}\right))}^{2}+{{(\Gamma }_{v}/2)}^{2}}$$

Here, the Lorentzian line shape reflects the resonant enhancement near *ω_p_* − *ω*_s_ = *Ω_v_.*

The peak gain *g*_0_ occurs at resonance (*ω_p_* − *ω*_s_ = *Ω_v_*):

(10) $$g_{0}=\frac{2\omega_{s}N}{c^{2}n_{p}n_{s}\varepsilon_{0}m_{e}\Omega_{v}\Gamma_{v}}$$

Then, the full SRS gain spectrum is written as:

(11) $$g_{s}\left(\omega \right)=g_{0}\frac{{{(\Gamma }_{v}/2)}^{2}}{{{(\omega_{p}-\omega_{s}-\Omega }_{v})}^{2}+{{(\Gamma }_{v}/2)}^{2}}$$

Secondly, the SRS-induced inter-channel energy transfer is discussed. When the wavelengths of 5G fronthaul and other protocols are simultaneously transmitted in the same optical fiber, the SRS interaction can be described as follows [22]:

(12) $$\frac{\partial P_{S}}{\partial z}+\frac{1}{v_{S}}\frac{\partial P_{S}}{\partial t}=\left(gP_{P}-\alpha \right)P_{S}$$

(13) $$\frac{\partial P_{P}}{\partial z}+\frac{1}{v_{P}}\frac{\partial P_{P}}{\partial t}=\left(-gP_{S}-\alpha \right)P_{P}$$

where $P_{S}$ is the power per Stokes wavelength, $P_{P}$ is the power per pump wavelength, and the group velocity of signal and pump signals is expressed by $v_{S}$ and $v_{P}$, respectively. g denotes the standard Raman gain coefficient ($g_{R}$) divided by the fiber effective area ($A_{\mathrm{eff}}$). Here, the power depletion on the pump channels is only considered and the Raman gain term ($gP_{P}P_{S}$) on the Stokes channels is neglected. Additionally, the time-dependent terms are disregarded because they are comparatively smaller. Therefore, Equations (12) and (13) can be rewritten as:

(14) $$\frac{\partial P_{S}}{\partial z}=-\alpha P_{S}$$

(15) $$\frac{\partial P_{P}}{\partial z}=\left(-gP_{S}-\alpha \right)P_{P}$$

Then, the SRS-induced power depletion ΔP under *N* Stokes channels can be represented as [23]:

(16) $$∆P=\sum_{i=1}^{N}\left(10\log_{10}e\frac{g_{Ri}}{A_{eff}}\right)P_{s}L_{eff}$$

where ${g_{R}}_{i}$ means the *i*-th channel’s Raman gain coefficient. It can be seen that the SRS-induced power depletion relies on the number of channels (*N*) and optical fiber length ($L_{eff}$).

Finally, the SRS-induces crosstalk is hashed out. In multi-wavelength systems, the SRS non-linearity impairment is complex; not only do shorter wavelengths transfer energy to longer wavelengths, but intermediate wavelengths also absorb energy from shorter wavelengths while transferring energy to longer wavelengths. For an M-wavelength system, the output power of each wavelength can be obtained by solving the M coupling equations, which can be written as following [24]:

(17) $$\frac{dP_{n}(z)}{dz}+\alpha P_{n}(z)+\frac{g^{\prime }\Delta f}{2A_{eff}}P_{n}(z)\sum_{m-1}^{M}(m-n)P_{m}(z)=0$$

where $P_{n}(z)$ is the power of the *N*-th wavelength and it is a function of the transmission distance *z*, α is the fiber attenuation, $g^{\prime }=\frac{dg_{R}}{df}$ denotes the slope of the Raman gain, Δf refers the channel spacing, $A_{eff}$ is the effective cross-sectional area of optical fiber, *m* and *n* represent the *M*-th and *N*-th wavelengths, respectively. In the derivation of Equation (17), the approximations are given in Table 1. Thus, achieving the worst crosstalk caused by SRS in WDM systems.

By solving Equation (17), $P_{n}(z)$ can be expressed as follows:

(18) $$P_{n}(z)=P_{no}J_{o}e^{-\alpha z}\exp[GJ_{o}(n-1)Z_{e}][{\sum_{m=1}^{M}P_{mo}e}^{GJ_{o}(m-1)Ze}{]}^{-1}$$

with

(19) $$G=\frac{g^{\prime }\Delta f}{2A_{\mathrm{eff}}}$$

(20) $$J_{o}=\sum_{m=1}^{M}P_{mo}$$

(21) $$Z_{e}=\frac{1-e^{-\alpha z}}{\alpha }$$

where *G* is the gain after averaging polarization, $P_{no}$ is the *N*-th wavelength power, $Z_{e}$ means effective gain length, and $J_{o}$ denotes the total power of the system. When all wavelengths have non-uniform channel spacing and the same input power ($P_{no}=P_{o}$), Equation (18) can be rewritten as:

(22) $$P_{n}(z)=NP_{o}e^{-\alpha z}\exp[(\frac{GJ_{o}Z_{e}}{2})(2n-N-1)]sinh(\frac{GJ_{o}Z_{e}}{2})sinh(\frac{NGJ_{o}Z_{e}}{2})$$

Then, the crosstalk of the shortest wavelength, which is the most serious, can be expressed as:

(23) $$XT(z)=[P_{o}e^{-\alpha z}-P_{1}(z)](P_{o}e^{-\alpha z}{)}^{-1}$$

When all wavelengths have the same channel spacing, the crosstalk impairment can be acquired from Equation (22) and is shown as follows:

(24) $$P_{n}\left(z\right)\;=1-N\exp[(-GNP_{o}Z_{e})(\frac{N-1}{2})]\sin h(\frac{NGP_{o}Z_{e}}{2})\sin h(\frac{N^{2}GP_{o}Z_{e}}{2}{)}^{-1}$$

In the limit of small inter-channel crosstalk, i.e., *P*_0_*GZ_e_* << 1, the Equation (24) can be rewritten as:

(25) $$XT(z)=\frac{N}{2}(N-1)P_{o}GZ_{e}$$

It can be seen that the SRS-induced crosstalk is related to the number of channels (*N*), input power ($P_{o}$), Raman gain ($g^{\prime }$), and effective gain length ($Z_{e}$).

## 3. Simulation Setup

**Figure 1:**
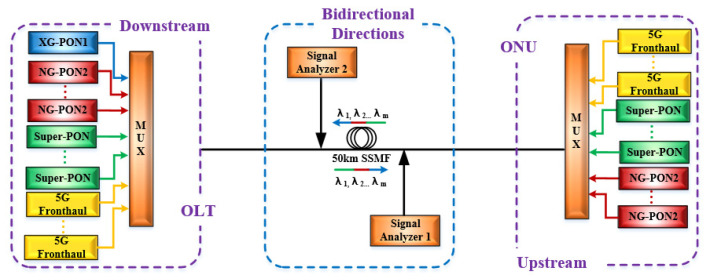
Simulation setup for measuring the SRS effect on the upstream and downstream wavelengths of the 5G fronthaul networks when NG-PON2, Super-PON, and XG-PON1 coexist with the 5G fronthaul upstream and downstream wavelengths.

In order to evaluate the effects of SRS on 5G fronthaul networks based on WDM-PON in the C-band, VPI transmission Maker simulation systems have been installed. Note that, in order to accurately acquire the effect of SRS non-linearity impairment on the 5G fronthaul networks, the Kerr effects shall be ignored. Figure 1 shows the most severe scenario when NG-PON2 and Super-PON (16 channels per direction [25]), and XG-PON1 (189.6 THz in the downstream direction), coexist with the upstream and downstream wavelengths of the 5G fronthaul networks, which is described as an example. The used parameters are given in Table 2. At the transmitter sides, 12 wavelengths working at 197.4 THz, 197.5 THz, 197.6 THz, 197.7 THz, 197.8 THz, 197.9 THz, 198 THz, 198.1 THz, 198.2 THz, 198.3 THz, 198.4 THz, and 198.5 THz are employed as the upstream optical carriers of the 5G fronthaul, whose channel spacing is 100 GHz. Meanwhile, 12 wavelengths operating at 184.6 THz, 184.7 THz, 184.8 THz, 184.9 THz, 185 THz, 185.1 THz, 185.2 THz, 185.3 THz, 185.4 THz, 185.5 THz, 185.6 THz and 185.7 THz are used as the downstream optical carriers of the 5G fronthaul, where the channel spacing is also 100 GHz. According to the 25G enhanced Common Public Radio Interface (eCPRI) in 5G fronthaul networks [26], 25 Gb/s NRZ, ODB and PAM4 signals are modulated on the upstream and downstream wavelengths through the use of Mach–Zehnder Modulators (MZMs) with a fixed chirp coefficient of 0 [27]. Considering the increasing demands for bandwidth, the transmission rate of 50 Gb/s is also taken into account [28]. The wavelengths are then amplified to 8 dBm per channel. After amplification, the upstream and downstream wavelengths of different protocols are coupled together by multiplexers (MUXs) with an insertion loss of 1.5 dB. For the optical fiber length, the typical 5G fronthaul and PON distances are 10 km and 20 km, respectively [29,30]. However, since the Super-PON protocol specifies an optical fiber length of 50 km [25], the coupled signal is transmitted over a 50-km optical fiber for bi-directional transmission, with an attenuation of 0.24 dB/km and chromatic dispersion of 16 ps/(nm∙km). Following the optical fiber transmission, the upstream and downstream wavelengths are directed into the “Signal Analyzers” modules. Note that the “Signal Analyzers” modules are also used to measure the power prior to the optical fiber transmission.

**Figure 2:**
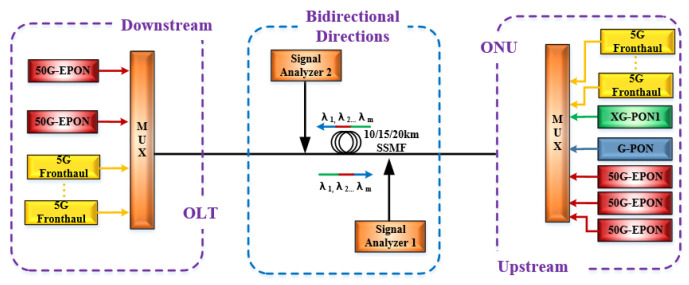
Simulation setup for measuring the SRS effect on the upstream and downstream wavelengths of the 5G fronthaul networks when XG-PON1, GPON, and 50G-EPON coexist with the 5G fronthaul upstream and downstream wavelengths.

To examine the impact of SRS on WDM-PON based 5G fronthaul networks in the O-band, a simulation system is set up as shown in Figure 2. The employed parameters are depicted in Table 3. Here, the most severe scenario is considered, where the XG-PON1, GPON, and 50G-EPON coexist with the 5G fronthaul wavelengths. At the Tx side of the upstream direction, 12 wavelengths working at 220.4 THz, 221.2 THz, 222 THz, 222.8 THz, 223.6 THz, 224.4 THz, 225.2 THz, 226 THz, 226.8 THz, 227.6 THz, 228.4 THz, and 229.2 THz are utilized as the 5G fronthaul upstream optical carriers of the 5G fronthaul, with a channel spacing of 800 GHz. Meanwhile, the XG-PON1 upstream wavelength (234.2 THz), GPON upstream wavelength (225.4 THz), and 50G-EPON upstream wavelengths (236.1 THz, 230.6 THz, and 226.8 THz) are transmitted simultaneously with the 5G fronthaul upstream wavelengths. On the Tx side of the downstream direction, 12 wavelengths operating at 228.9 THz, 229.7 THz, 230.5 THz, 231.3 THz, 232.1 THz, 232.9 THz, 233.7 THz, 234.6 THz, 235.4 THz, 236.2 THz, 237.1 THz, and 237.9 THz are used as the downstream optical carriers of the 5G fronthaul, with a channel spacing of 800 GHz. The 50G-EPON downstream wavelengths (223.4 THz and 220.8 THz) are transmitted alongside the 5G fronthaul downstream wavelengths. The upstream and downstream wavelengths of 5G fronthaul networks in O-band are modulated by 25 Gb/s and 50 Gb/s NRZ, ODB and PAM4 signals through MZMs. Afterwards, the upstream and downstream wavelengths are amplified to 8 dBm and coupled together by multiplexers (MUXs), where the insertion loss is 1.5 dB. Then, the coupled wavelengths are sent into a 10/15/20-km optical fiber for bi-direction transmission, whose attenuation and chromatic dispersion are 0.34 dB/km and 2 ps/(nm∙km), respectively. In order to calculate the SRS-induced power changes, the “Signal Analyzers” modules are adopted for measuring the optical spectrums of upstream and downstream wavelengths before and after optical fiber transmission.

## 4. Results

### 4.1. SRS-Induced Power Changes in C-Band Allocation Scheme

**Figure 3:**
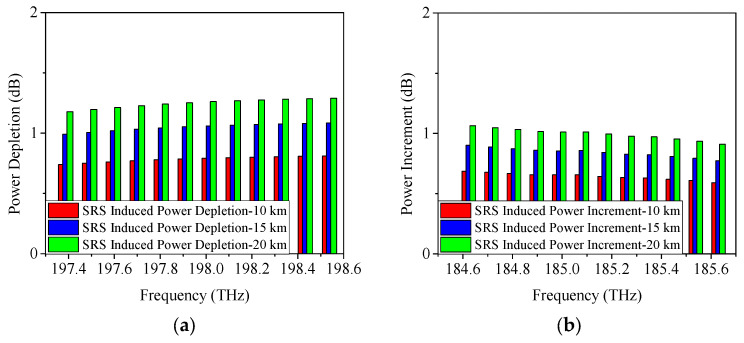
Measured power changes in 5G fronthaul wavelengths due to SRS for (**a**) upstream and (**b**) downstream channels, with a modulation format of 25 Gb/s NRZ.

**Figure 4:**
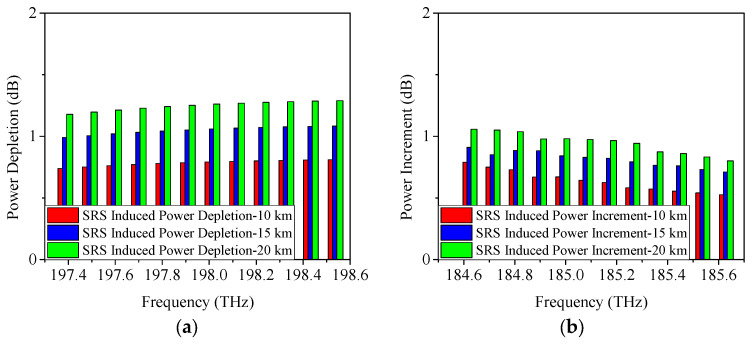
Measured power changes in 5G fronthaul wavelengths due to SRS for (**a**) upstream and (**b**) downstream channels, with a modulation format of 25 Gb/s ODB.

**Figure 5:**
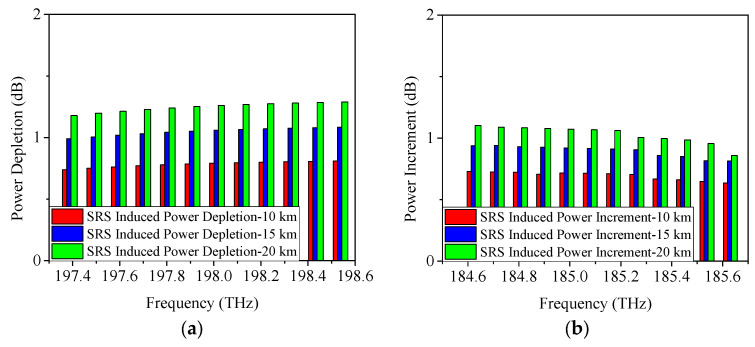
Measured power changes in 5G fronthaul wavelengths due to SRS for (**a**) upstream and (**b**) downstream channels, with a modulation format of 25 Gb/s PAM4.

As discussed in Section 2, SRS non-linearity impairment results in power fluctuations among the transmitted wavelengths. To further analyze the proposed scheme, Figure 3, Figure 4 and Figure 5 display the SRS-induced power changes using 25 Gb/s NRZ, ODB, and PAM4 modulation formats, respectively, when only the upstream and downstream wavelengths of the 5G fronthaul are transmitted through the optical fiber. Here, the power depletion/increment refers the difference in power between the spectrum subject to attenuation and SRS, and subject to attenuation only, i.e., the difference between the power before fiber optic transmission minus the fiber optic attenuation and the signal power after fiber optic transmission is the SRS-induced power depletion/increment. It is clear that the 5G fronthaul upstream wavelengths operate as pump channels and experience SRS-induced power depletion because they have higher frequencies. It is important to note that Channel 12 (198.5 THz) of the 5G fronthaul upstream wavelengths, being the highest frequency, experiences the most severe SRS non-linearity impairment. Consequently, it is used as a representative example to demonstrate the power depletion caused by SRS non-linearity impairment. From Figure 3a, it can be seen that, when the transmission distance is 10 km and the modulation format is 25 Gb/s NRZ, the SRS-induced power depletion is 0.8 dB, while, with the increase of the transmission distance, the interaction time between the wavelengths will last longer, making the SRS non-linearity impairment more severe. Therefore, the SRS non-linearity impairment results in a 1.1-dB power penalty at a transmission distance of 15 km. Even worse, a 1.3-dB power depletion is occurred on Channel 12 (198.5 THz) with the transmission distance increased to 20 km. The obtained simulation results are in accordance with the theoretical part of Section 2, i.e., the SRS-induced power depletion relies on the optical fiber length. In contrast, since the 5G fronthaul downstream wavelengths are lower in frequency, they act as Stokes channels and experience an increase in power due to SRS-induced effects. The power increase of Channel 1 (184.6 THz), the lowest frequency among the 5G fronthaul downstream wavelengths, is used as an example to illustrate this effect. After 10 km optical fiber transmission, the SRS-induced power increment is 0.7 dB as depicted in Figure 3b. Similar to the above-mentioned results of SRS-induced power depletion, when the transmission distance is increased to 15 km/20 km, the SRS-induced power increment runs to 0.9 dB/1.1 dB.

When the modulation format is 25 Gb/s ODB, the obtained power changes caused by SRS are given in Figure 4. From Figure 4a, it is shown that when the transmission distance is 10 km, the power depletion occurred on Channel 12 is measured as 0.8 dB, which is similar to the result of 25 Gb/s NRZ. Moreover, the SRS-induced power depletion is 1.1 dB/1.3 dB when the transmission distance is 15 km/20 km, which is also similar to the above-mentioned result of 25 Gb/s NRZ. Figure 4b demonstrates the SRS-induced power increment when the modulation format is 25 Gb/s ODB. It can be seen that, under the conditions of 10 km, 15 km, and 20 km link length, the SRS-induced power increments are 0.7 dB, 0.9 dB, and 1.1 dB, respectively.

When the modulation format is 25 Gb/s PAM4, the achieved power depletion/increment induced by SRS are indicated in Figure 5. Similarly, the SRS-induced power depletions on Channel 12 (198.5 THz) are also 0.8 dB, 1.1 dB, and 1.3 dB, respectively. And the SRS-induced power increments on Channel 1 (184.6 THz) are 0.7 dB, 0.9 dB, and 1.1 dB, respectively. Based on the results obtained using 25 Gb/s NRZ, ODB, and PAM4 modulation formats, it can be concluded that SRS-induced power changes are minimally affected by the modulation format. In other words, the power changes due to SRS remain nearly consistent across different modulation formats.

**Figure 6:**
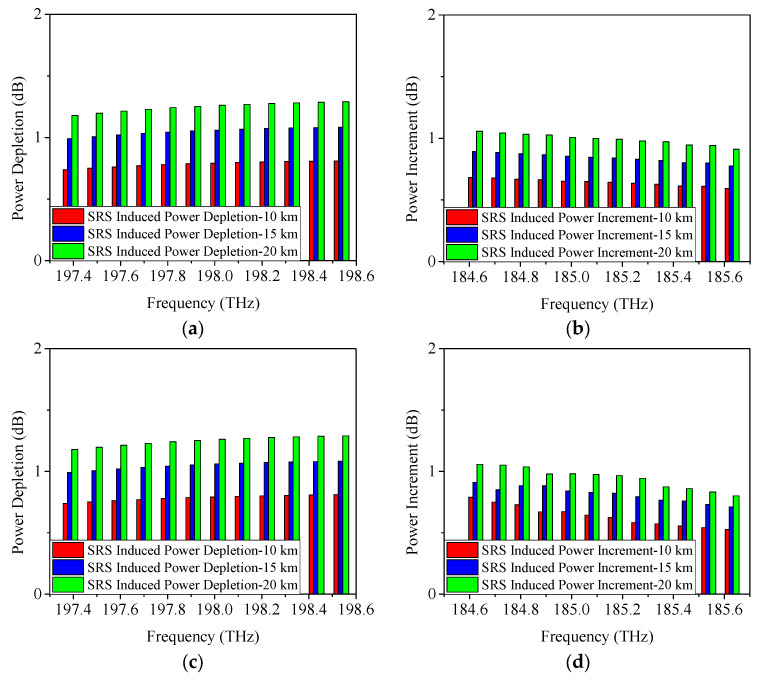

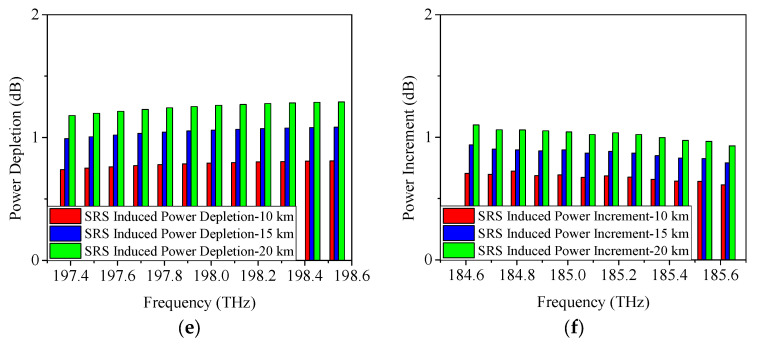
Measured power changes in 5G fronthaul wavelengths due to SRS for (**a**,**c**,**e**) upstream (**b**,**d**,**f**) downstream channels, with modulation formats of 50 Gb/s (**a**,**b**) NRZ, (**c**,**d**) ODB and (**e**,**f**) PAM4, respectively.

Considering the continuous improvement of communication capacity in the future, the transmission rate of 5G fronthaul upstream and downstream wavelengths is increased to 50 Gb/s per channel and the acquired power changes are represented in Figure 6. It can be seen that, regardless of whether the modulation format is NRZ, ODB, or PAM4, the power depletion of Channel 12 due to SRS non-linearity impairment are 0.8 dB, 1.1 dB, and 1.3 dB, respectively. And the power increments of Channel 1 are 0.7 dB, 0.9 dB, and 1.1 dB, respectively. Therefore, combining the conclusions above for a 25 Gb/s transmission rate, it can be inferred that SRS-induced power changes are largely independent of both modulation formats and modulation rates.

**Figure 7:**
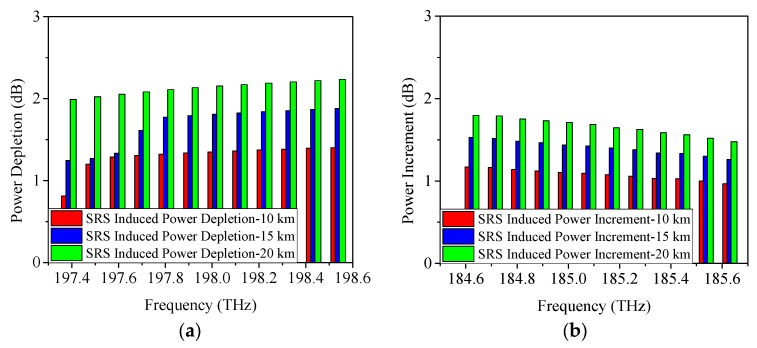
Measured power changes in 5G fronthaul wavelengths due to SRS for (**a**) upstream and (**b**) downstream channels, with a modulation format of 25 Gb/s NRZ.

Subsequently, Figure 7 illustrates the SRS-induced power changes when the 5G fronthaul wavelengths (25 Gb/s NRZ signals) are transmitted simultaneously with NG-PON2 in the optical fiber. It can be seen that, with the increase of the transmitted wavelengths, the SRS non-linearity impairment becomes more severe as indicated in Figure 7a. The SRS-induced power depletion on Channel 12 (198.5 THz) is measured as 1.4 dB when the transmission distance is 10 km, which is 0.6 dB higher than that of the transmission case of only 5G fronthaul upstream and downstream wavelengths. More severely, when the transmission distance extends to 20 km, a 2.2-dB power depletion is caused by SRS non-linearity impairment, which is 0.9 dB higher than that of the transmission case of only 5G fronthaul upstream and downstream wavelengths. It is consistent with the conclusions obtained from the theoretical analysis (Section 2), i.e., the SRS-induced power depletion is related to the number of channels.

**Figure 8:**
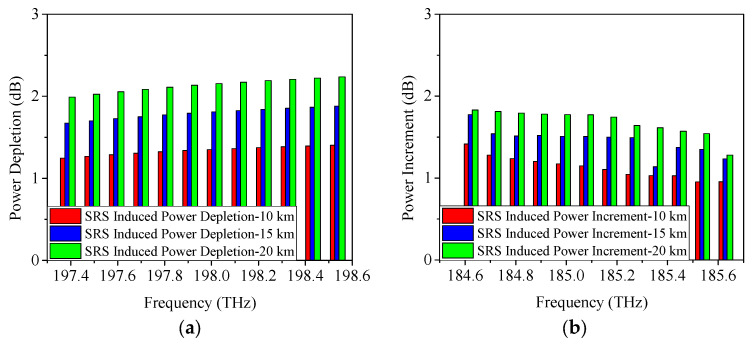
Measured power changes in 5G fronthaul wavelengths due to SRS for (**a**) upstream and (**b**) downstream channels, with a modulation format of 25 Gb/s ODB.

**Figure 9:**
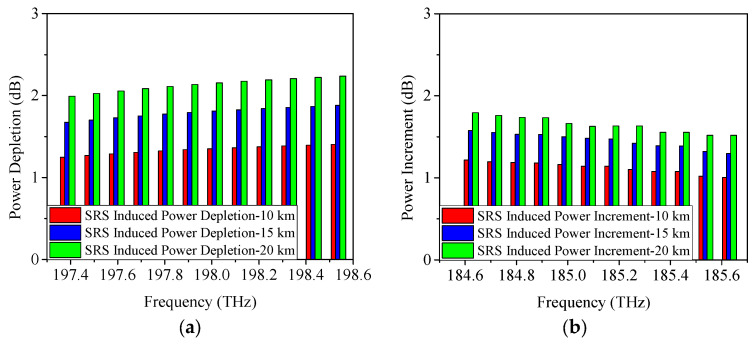
Measured power changes in 5G fronthaul wavelengths due to SRS for (**a**) upstream and (**b**) downstream channels, with a modulation format of 25 Gb/s PAM4.

Coming to the SRS-induced power increment, a 1.8-dB power increment is obtained on Channel 1 (184.6 THz) of the 5G fronthaul downstream wavelengths when the transmission distance is 20 km, which is 0.7 dB higher than that of the transmission case of only 5G fronthaul upstream and downstream wavelengths. Similar power change results on Channel 12 can be achieved when the modulation format is ODB or PAM4 (25 Gb/s) as shown in Figure 8 and Figure 9, which, again, validate the above-mentioned conclusion.

**Figure 10:**
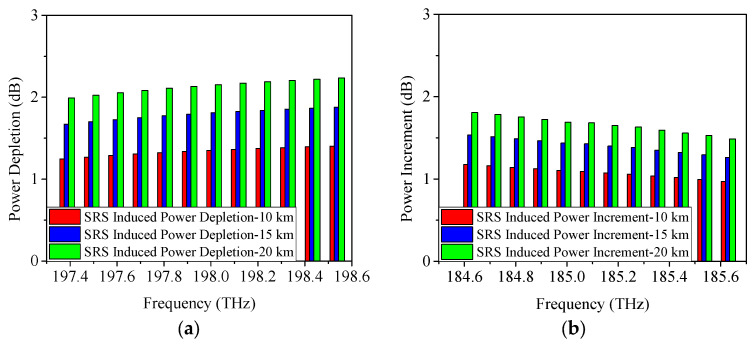

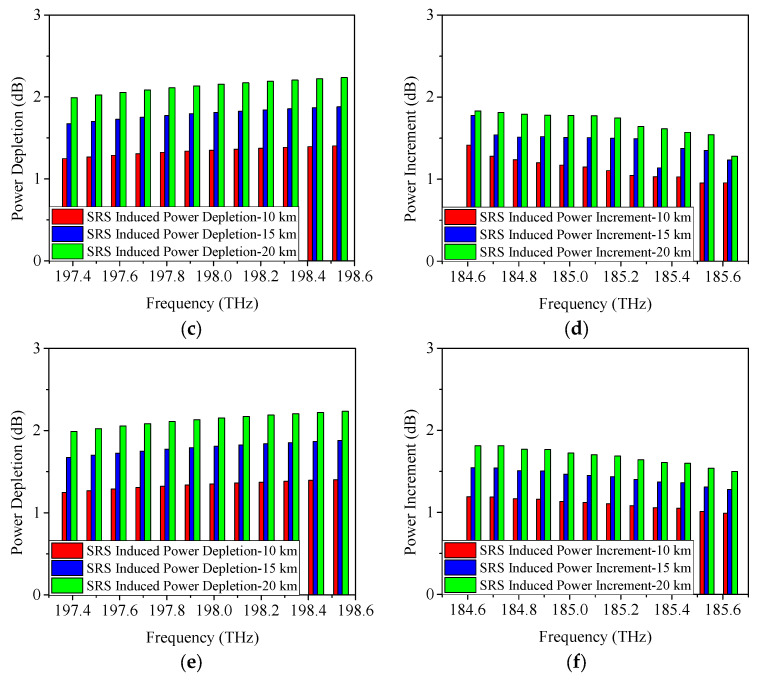
Measured power changes in 5G fronthaul wavelengths due to SRS for (**a**,**c**,**e**) upstream (**b**,**d**,**f**) downstream channels, with modulation formats of 50 Gb/s (**a**,**b**) NRZ, (**c**,**d**) ODB, and (**e**,**f**) PAM4, respectively.

When boosting the single wavelength rate to 50 Gb/s, the obtained power changes caused by SRS are illustrated in Figure 10. According to the previously obtained conclusion, the power penalty caused by SRS non-linearity impairment is not significantly related to the modulation formats and modulation rates, i.e., the power penalty induced by SRS non-linearity impairment for 50 Gb/s NRZ, ODB, and PAM4 modulation formats should be 1.4 dB, 1.9 dB, and 2.2 dB, respectively, which is indeed the case with the results obtained from the simulations (shown in Figure 10).

**Figure 11:**
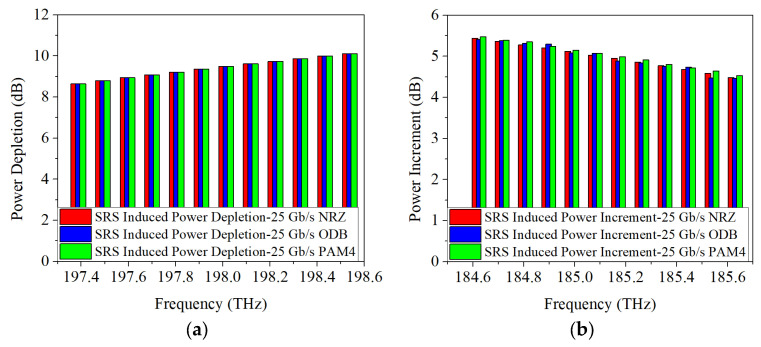
Measured power changes in 5G fronthaul wavelengths due to SRS for (**a**) upstream and (**b**) downstream channels, with modulation formats of 25 Gb/s NRZ, ODB and PAM4, respectively.

**Figure 12:**
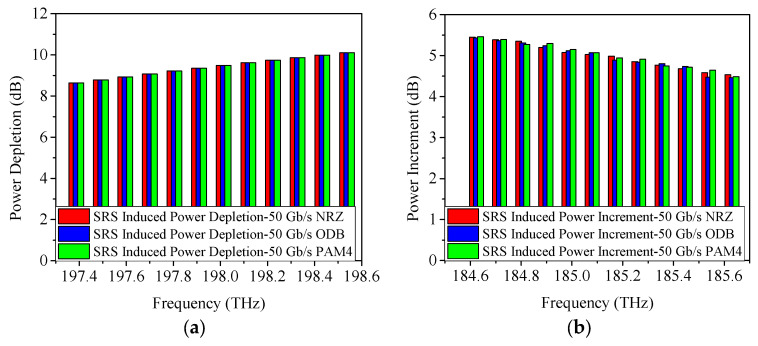
Measured power changes in 5G fronthaul wavelengths due to SRS for (**a**) upstream and (**b**) downstream channels, with modulation formats of 50 Gb/s NRZ, ODB, and PAM4, respectively.

Finally, the power changes resulting from SRS non-linearity impairment are illustrated in Figure 11, where the 5G fronthaul wavelengths coexist with NG-PON2 and Super-PON (16 channels per direction), and XG-PON1 (189.6 THz in the downstream direction). As a result, the SRS non-linearity impairment involves additional wavelengths, resulting in a greater degree of SRS-induced power changes. From Figure 11a, it can be seen that more than 10.1 dB power depletion is generated by the SRS non-linearity impairment on Channel 12 (198.5 THz) when the transmission distance is 50 km, which will seriously affect the power and quality of the transmitted wavelengths, thus threating the loss budget of the 5G fronthaul networks. Figure 11b shows that the SRS-induced power increment on Channel 1 (184.6 THz) is 5.4 dB. Additionally, when the transmission rate is increased to 50 Gb/s, the SRS-induced power changes remain nearly identical to those observed in the 25 Gb/s transmission cases, as shown in Figure 12.

### 4.2. SRS-Induced Power Changes in O-Band Allocation Scheme

**Figure 13:**
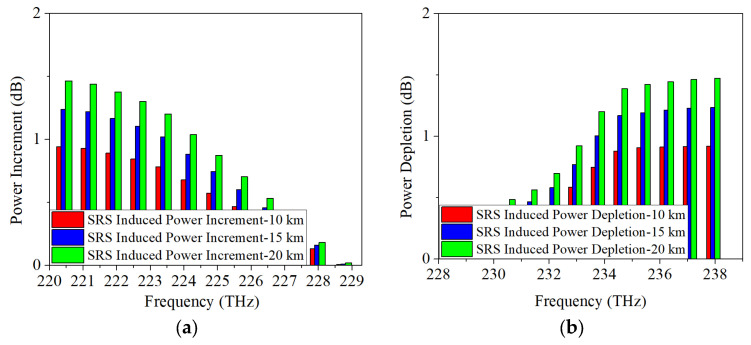
Measured power changes in 5G fronthaul wavelengths due to SRS for (**a**) upstream and (**b**) downstream channels, with a modulation format of 25 Gb/s NRZ.

**Figure 14:**
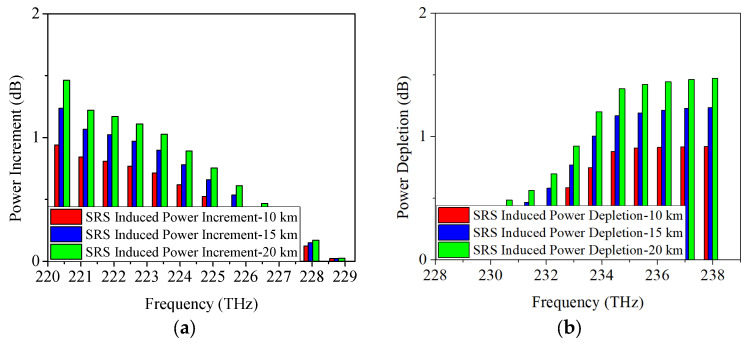
Measured power changes in 5G fronthaul wavelengths due to SRS for (**a**) upstream and (**b**) downstream channels, with a modulation format of 25 Gb/s ODB.

**Figure 15:**
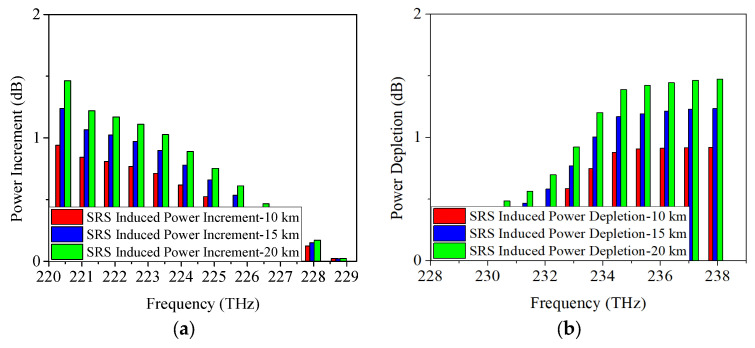
Measured power changes in 5G fronthaul wavelengths due to SRS for (**a**) upstream and (**b**) downstream channels, with a modulation format of 25 Gb/s PAM4.

**Figure 16:**
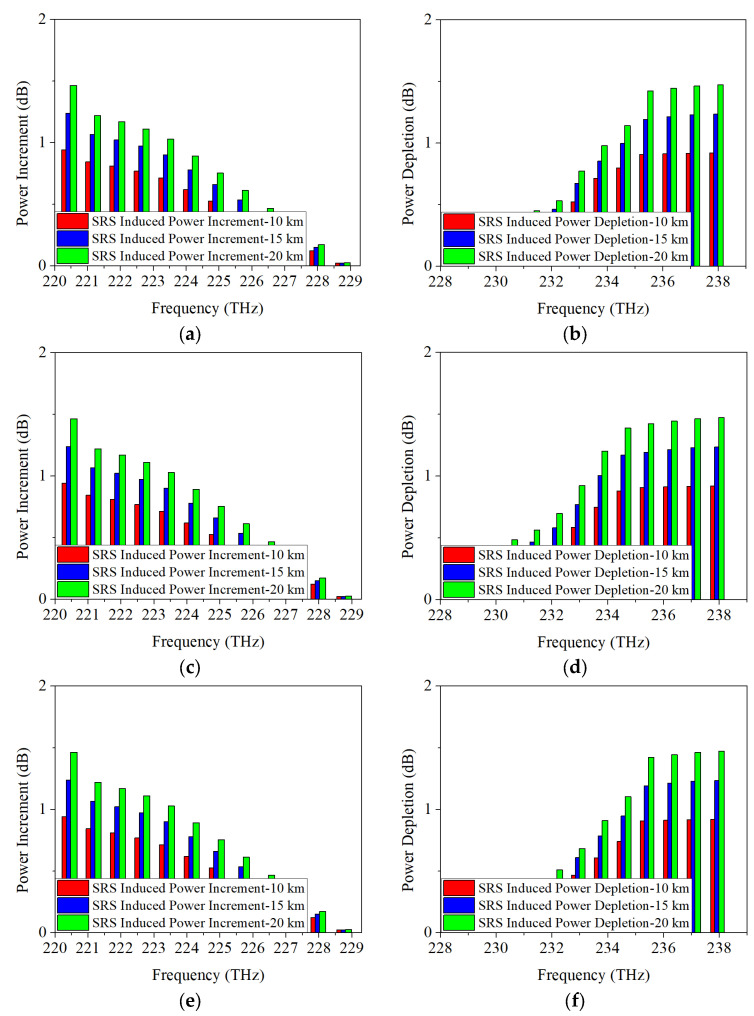
Measured power changes in 5G fronthaul wavelengths due to SRS for (**a**,**c**,**e**) upstream (**b**,**d**,**f**) downstream channels, with modulation formats of 50 Gb/s (**a**,**b**) NRZ, (**c**,**d**) ODB, and (**e**,**f**) PAM4, respectively.

When the 5G fronthaul networks use O-band for transmission, the SRS-induced power changes adopting NRZ, ODB, and PAM4 modulation formats are represented in Figure 13, Figure 14 and Figure 15. Here, the most complex scenario is considered, where the XG-PON1, GPON, and 50G-EPON coexist with the 5G fronthaul wavelengths. The higher frequencies of the downstream wavelengths will transmit some of their energy to the lower frequencies of the upstream wavelengths. As the highest/lowest frequency, Channel 12 (237.9 THz)/Channel 1 (220.4 THz) of the downstream/upstream wavelength is selected for investigating the SRS-induced power depletion/increment. From Figure 13a,b, it shows that a 1.5-dB/1.5-dB power increment/depletion is caused by the SRS non-linearity impairment using 25 Gb/s NRZ. When the modulation formats are ODB and PAM4, similar results can be obtained as represented in Figure 14 and Figure 15, i.e., SRS-induced power depletion is 1.5 dB, and SRS-induced power increment is also 1.5 dB. The results obtained after increasing the single-wavelength transmission rate to 50 Gb/s are almost consistent with those obtained at the 25-G cases, as shown in Figure 16.

## 5. Conclusions

In this paper, the SRS-induced power changes are investigated in WDM-PON-based 5G fronthaul networks, where both the C-band and O-band are adopted for the wavelength allocation schemes, and 25 Gb/s or 50 Gb/s NRZ, ODB, and PAM4 are employed as modulation formats. In the C-band scenario, when only the upstream and downstream wavelengths of the 5G fronthaul are transmitted through the optical fiber, the SRS-induced power depletion amounts to 1.3 dB. However, when 5G fronthaul wavelengths coexist with NG-PON2, Super-PON, and XG-PON1 wavelengths in the future hybrid fiber and radio access network, the power depletion is increased to 10.1 dB, significantly impacting the loss budget requirements of the 5G fronthaul networks. In the O-band scenario, the SRS-induced power depletion reaches a maximum of 1.5 dB. These results shall offer valuable insights for optimizing wavelength allocation in 5G fronthaul networks.

## Figures and Tables

**Table 1:** The used assumptions in the derivation of Equation (17).

Serial Number	Approximate Condition
1	All channels lie within the linear portion of the Raman gain profile and are equally separated in the spectral domain
2	The triangular approximation is employed to the Raman gain curve (i.e., the Raman gain is assumed to vary linearly)
3	$\frac{\omega_{n}}{\omega_{m}}\approx 1$
4	The walk-off effect between wavelengths is ignored

**Table 2:** Simulation parameters for investigating the SRS in 5G fronthaul networks using C-band.

Parameters	Value
Wavelengths (5G fronthaul upstream)	197.4~198.5 THz
Wavelengths (5G fronthaul downstream)	184.6~185.7 THz
Channel Spacing	100 GHz
Modulation Format	NRZ, ODB, PAM4
Modulation Rate	25 Gb/s, 50 Gb/s (5G fronthaul), 10 Gb/s (NG-PON2, Super-PON and XG-PON1)
Fiber Attenuation	0.24 dB/km
MUX and DEMUX Insertion Loss	1.5 dB
Chirp Coefficient	0
Out Power	8 dBm/channel, 12 dBm/channel (for Super-PON)
Chromatic Dispersion of Fiber	16 ps/(nm·km)
Fiber Length	10 km, 15 km, 20 km, 50 km (for Super-PON)

**Table 3:** Simulation parameters for studying the SRS in 5G fronthaul networks using O-band.

Parameters	Value
Wavelengths (5G fronthaul upstream)	220.4~229.2 THz
Wavelengths (5G fronthaul downstream)	228.9~237.9 THz
Channel Spacing	800 GHz
Modulation Format	NRZ, ODB, PAM4
Modulation Rate	25 Gb/s, 50 Gb/s (5G fronthaul), 25 Gb/s (50G-EPON), 10 Gb/s (XG-PON1), 1 Gb/s (GPON)
Fiber Attenuation	0.34 dB/km
MUX and DEMUX Insertion Loss	1.5 dB
Chirp Coefficient	0
Out Power	8 dBm/channel
Chromatic Dispersion	2 ps/(nm·km)
Fiber Length	10 km, 15 km, 20 km

## Data Availability

The data are available from the corresponding author on reasonable request.
